# Shaping ability of four root canal instrumentation systems in simulated 3D-printed root canal models

**DOI:** 10.1371/journal.pone.0201129

**Published:** 2018-08-01

**Authors:** David Christofzik, Andreas Bartols, Mahmoud Khaled Faheem, Doreen Schroeter, Birte Groessner-Schreiber, Christof E. Doerfer

**Affiliations:** 1 Clinic for Conservative Dentistry and Periodontology, School for Dental Medicine, Christian-Albrechts-University Kiel, Kiel, Germany; 2 Dental Academy for Continuing Professional Development Karlsruhe, Karlsruhe, Germany; Ecole Normale Supérieure de Lyon, FRANCE

## Abstract

**Introduction:**

The aim of this study was to compare the shaping ability of four root canal preparation systems in newly developed 3D-printed root canal models.

**Materials and methods:**

For this study, 1080 3D-printed acrylic resin blocks with nine different root canal configurations were produced. They were prepared with Reciproc R25 (#25), F6 SkyTaper (#25 and #30) F360 (#25 and #35) and One Shape (#25) (N = 30 per system). Pre- and post-instrumentation images were superimposed for evaluation of the centering ratio of the different systems. Ledges, instrument fractures and preparation times were also recorded. Analysis of variance (ANOVA) and post-hoc Tukey tests were conducted, comparing the mean canal centering ratios and the mean preparation times.

**Results:**

There were significant differences between all systems regarding the centering ratios in the different root canal configurations (ANOVA p < 0.001). The root canal configuration had considerable effect on the centering ratio of the instruments. The best overall mean centering ratios were achieved with F6 SkyTaper #25 instruments especially in canal configurations with big curvature angles and radii, while F360 #35 was least centered especially in canals with small curvature angles and radii. Most ledges occurred with OneShape, while it was the significantly (p < 0.001) fastest preparation system (86.7 s (SD 13.53)) and Reciproc the significantly (p < 0.001) slowest (103.0 s (SD 20.67)).

**Conclusion:**

3D-printed root canals are suitable to produce challenging canal configurations and to investigate the limitations of root canal instruments. We found that all instruments caused canal transportations. However, F6 SkyTaper #25 files had better overall centering ratios than the other instruments. In canal configurations with small curvature radii, the centering ratio of some instruments is low and the probability for ledges is increased.

## Introduction

There is no standard protocol to compare the shaping ability of different root canal instruments in a reproducible way, even though there are plenty of publications describing different in-vitro methods to evaluate such instruments [1–14]. These publications can be divided into publications performed on extracted teeth [2–6, 10, 12, 13] or on simulated root canals in resin blocks [1, 7–9, 11, 14, 15]. While some researchers advocated root canals in extracted teeth for the more realistic situation with natural dentine properties [16], others advocated the advantage of high standardization of simulated artificial root canals [15, 16]. Because natural teeth are not standardized, and each root canal has an individual course, this leads to individual measurements for every prepared root canal with different techniques like measurements with 2- or 3-dimensional X-ray technologies [3, 5, 17]. In contrast to that, transparent simulated root canal models give the possibility to superimpose pre- and post-instrumentation images [14, 15]. Afterwards measurements can be made at different levels of the root canal to calculate centering ratios [18] and measure the prepared canal space [15]. There are two main concerns regarding the precision of this method. First, measurement points were mostly placed manually and are therefore prone to subjectivity to a certain degree. Newer methods that were described in literature use digital technology to evaluate morphological changes pre- and post-instrumentation of standardized root canals, which reduces this kind of subjectivity substantially [19–21]. Second, conventionally manufactured simulated root canal models have production-related deviations, so that even these models are not exactly identical and lack standardization [22].

By using stereolithographic materials in a 3D-printing process, highest fabrication accuracy and standardization of these models can be obtained. Therefore it is now possible to produce and use highly accurate models that share the same minute details, which standardizes the comparison process [23].Moreover, this includes the option of fabricating various challenging root canal configurations, such as C- or J- shaped canals [24], with high reproducibility and to investigate systematically the effects of different preparation techniques under such standardized conditions.

The newest developments in root canal preparation led to the introduction of instrument systems with a much reduced set of files up to single-file systems. The single-file systems can be divided into reciprocating and rotary systems. Multiple-file systems are normally rotary systems.

One representative of single-file reciprocating systems is Reciproc (VDW, Munich, Germany). Reciproc files are made of M-wire nickel titanium and are designed for single use. The Reciproc system is used in the single-length technique. The R25 file has a diameter of ISO 25 at the instrument tip and a taper of 0.08. All Reciproc instruments have the specified taper within the first three millimeters of the file beginning at the instrument tip. The taper is regressive towards the shaft. The distance between the cutting edges is progressive. This design is intended to increase the removal of the debris and the efficiency of the cutting performance. Reciproc files have a non-cutting instrument tip. The cross-section of the instrument is S-shaped with two active cutting edges.

A representative of a single-file rotary system is OneShape (Micro-Mega, Besançon Cedex, France). The OneShape file system consists of one single file for rotary preparation of the entire root canal. The file has a diameter of ISO 25, a taper of 0.06 and a variable, asymmetrical cross-section. The instrument has a S-shaped cross section near the shaft, a diamond-shaped cross-section in the middle part and a triangular cross-section near the tip. The number of cutting edges is variable (two or three) because of the asymmetric cross-section. The instrument tip is non-cutting.

An example for very reduced multiple file rotaries are F360 instruments (Komet Dental, Gebr.Brasseler, Lemgo, Germany). F360 consists of a sequence of only two consecutive files of ISO sizes 25 and 35 with a taper of 0.04. The files have a S-shaped cross-section and are used in single-length technique. The instruments have two active cutting edges and a non-cutting instrument tip.

F6 SkyTaper is an example for a single-length rotary multiple-file system and has five different file sizes (ISO 20, 25, 30, 35 and 40). All instruments have a continuous taper of 0.06 throughout the entire working part. The files also have a S-shaped cross-section with two active cutting edges. The instruments have a non-cutting tip.

All four aforementioned instrument systems were already investigated in extracted teeth models in different studies that came to the conclusion that all instruments respected the original canal curvature well and were safe to use [25–27]. But to the best knowledge of the authors all four systems have never been directly compared in the same experimental setting.

Therefore, the purpose of this study was to compare the shaping ability of the four root canal preparation systems (Reciproc, F6 SkyTaper, F360 and OneShape) using newly developed 3D-printed root canal models with various, but highly standardized root canal anatomies.

## Materials and methods

### Root canal models

**Fig 1:**
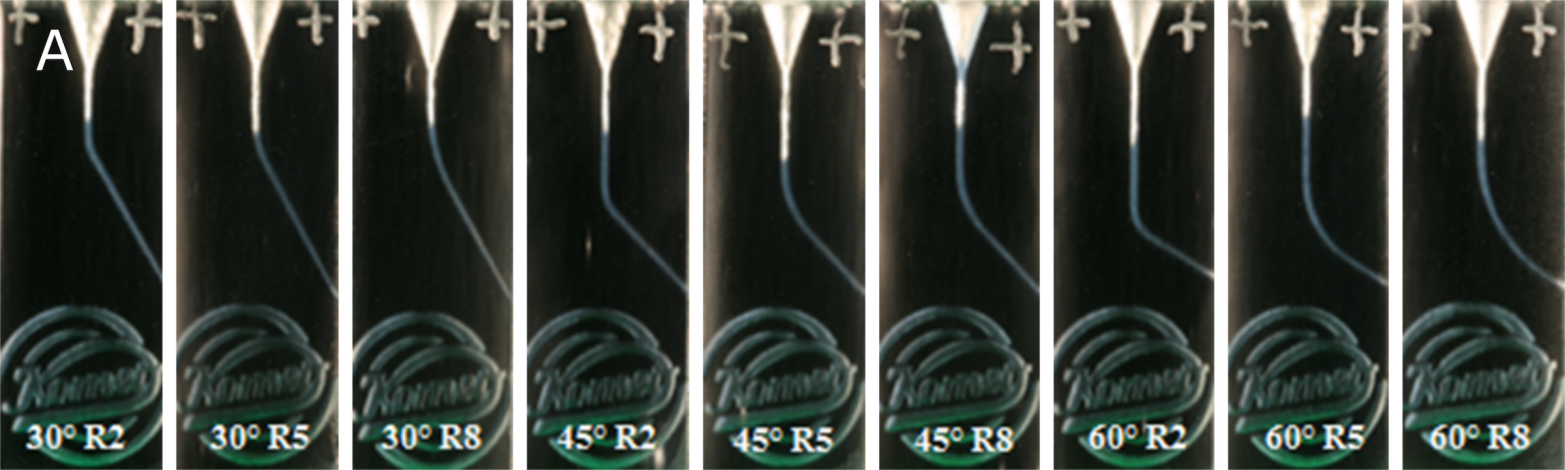
Shape of the different root canal configurations used in this study.

For this study, 1080 acrylic resin blocks with simulated root canals with different canal configurations were produced. The blocks were printed with acrylic resin (VeroClear-RGD810, Stratasys, Eden Prairie, USA) with the 3D precision printer Objet30 Pro (Stratasys, Eden Prairie, USA). This printer can print 16 μm thick layers of acrylic resin with a precision of 100 μm. During and after the printing process the printer heads for predefined control points to measure the dimensional precision of the print and thereby self-controls the quality of the printed product. Nine different root canal configurations were produced out of the possible combinations of three different curvature angles (60°, 45° and 30°) and three different curvature radii (8 mm, 5 mm and 2 mm) according to Schneider’s approach [28] (Fig 1). All root canals had a diameter of 0.15 mm and a length of 18 mm. Also, a pilot study was performed with a production of ten 3D-printed blocks that were then examined thoroughly and found to be identical.

### File systems and canal preparation

In this study, one reciprocating (Reciproc R25, #25) and three different rotary nickel-titanium (NiTi) root canal preparation systems (F6 SkyTaper #25 and #30, F360 #25 and #35 and OneShape #25) were used.

All instrument systems were used in the single length preparation approach. The canal preparations were performed with a Reciproc silver motor (VDW, Munich, Germany) with the correct settings for each file system according to the manufacturer’s instructions.

The F360 and F6 SkyTaper systems were used in sequence (ISO 25 and 35 and ISO 25 and 30) in the same root canal model. The reciprocating instrumentations were performed without further glide path preparation according to the manufacturer’s instructions for Reciproc [29].

Thirty specimens of the nine different simulated root canal anatomies were prepared with every file system. The coronal flaring and the glide path in the different models were already incorporated by the standardized 3D printing process.

Before root canal preparation the patency of the canals was checked with an ISO 10 K-file, exept in cases that were prepared with Reciproc. All root canals were 18 mm long and the working length was set to 17 mm. All files in this study were used to prepare only one root canal. The instruments were used in a pecking motion. After three motions, the instrument was removed, the canal was rinsed with 2 ml ethanol and patency was checked with an ISO 10 K-file. When the file reached workling length it was immediately removed and the canal was finally thoroughly rinsed until the debris was completely removed.

### Analysis of root canal preparation

**Fig 2:**
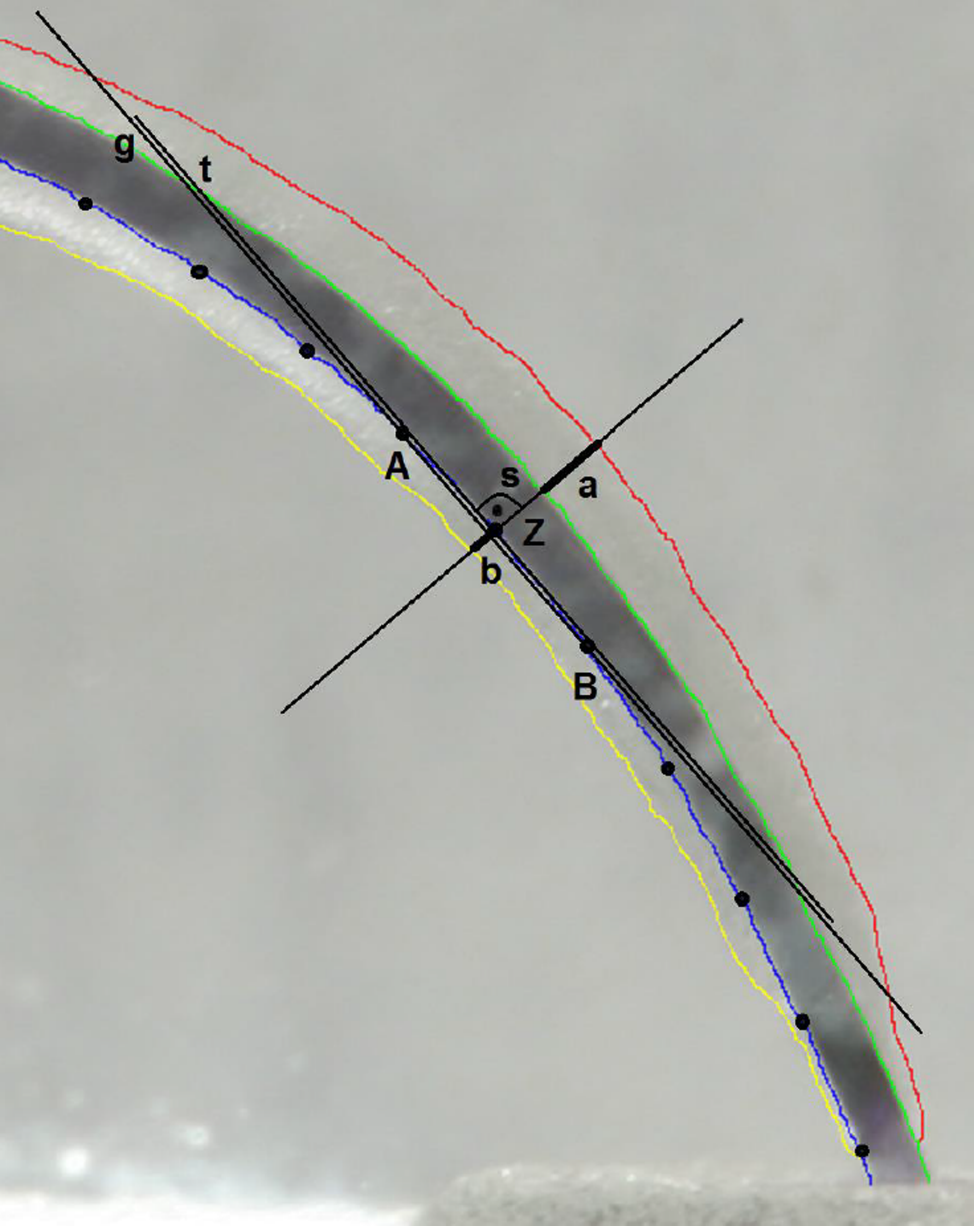
Schematic illustration of the function of the experimental software used to evaluate the centering ratios of the root canal preparations. Superimposed images with traced outlines of un-instrumented (green and blue lines) and instrumented (red and yellow lines) root canals with software assisted construction of tangents and perpendiculars crossing the outlines of the superimposed root canals. **Z**: current measuring point, **A** & **B**: neighbor auxiliary points at intervals of 15 pixels to Z, **g**: straight line constructed crossing **A** & **B**, **t**: tangent constructed intersecting **Z** by parallel shift to **g**, **s**: perpendicular intersecting **g** and **t** in **Z**, **a** & **b**: amount of canal wall removal detected by the software.

For the reproducible evaluation of the preparation results, all resin blocks were photographed before instrumentation. All blocks were fixed on an exactly fitting template connected to a repro stand (Nikon Repro-Copy Outfit PF4, NIKON Corporation, Tokyo, Japan). The standardized photographs were taken with a remote controlled camera (Canon EOS D400, Tokyo, Japan) with a macro objective (Canon EF 100 mm/ 1:2.8 USM, Tokyo, Japan) before and after root canal preparation. For the F360 and F6 SkyTaper systems a photograph was taken for intermediate analysis after the canal was prepared with the #25 instrument. Then the second instrument was used and the final evaluation of the prepared canal was done. Therefore the corresponding pre- and post-instrumentation pictures were superimposed with Adobe Photoshop CS5.1 (Adobe Systems, San José, USA). The outlines of the canals were traced with a specially developed experimental software tool, with which the spaces between the unprepared and prepared root canals could be measured automatically (Fig 2).

Measurements of the removed canal wall on the inner and outer surface were taken on the entire length of the canal at intervals of 15 pixels beginning at the most apical extent of the canal preparation ending at the canal orifice. Points A and B were the neighbor measuring points of the current measuring point Z. The straight line g was constructed through point A and B (Fig 2). After that a parallel line t was constructed to g as a tangent intersecting measuring point Z. Then the perpendicular line s was constructed to the tangent t that intersects Z. The amounts of the removed spaces a and b were set into relation and resulted in a local centering ratio. After that the mean centering ratio of the complete canal was calculated out of all local centering ratios. At the end a characteristic centering ratio resulted for every root canal anatomy and instrument system. An ideally centered root canal preparation would have the value of 1.0.The smaller the value, the greater the difference in the removal rate between the inner and outer curvatures of the canal.

Moreover, the time needed for root canal preparation including all rinsing and recapitulations between the rotary instruments was recorded. Procedural errors like ledges and instrument fractures were documented.

### Statistical analysis

For statistical analysis GraphPadPrism 6 (GraphPad Software, Inc., La Jolla, CA, USA) was used. Analysis of variance (ANOVA) and post-hoc Tukey-HSD tests were performed to compare the different groups regarding the mean canal centering ratios and the mean preparation times. The alpha-type error was set to 0.05. The results were transferred into error bar plots with symbols presenting the mean centering ratio and whiskers presenting the minimum and maximum centering ratios.

## Results

### Evaluation of the different preparation systems

**Fig 3:**
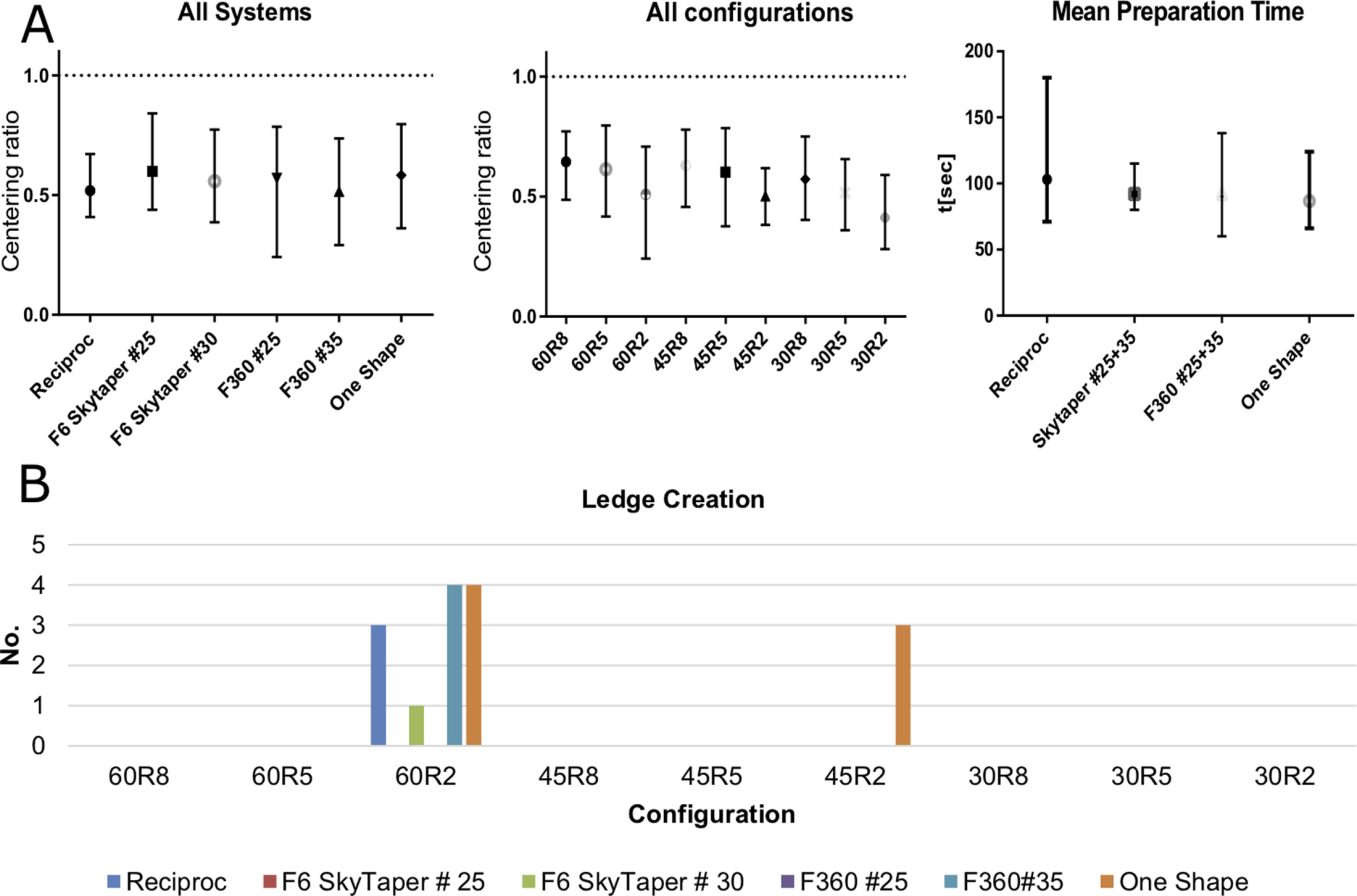
**A** Left: Error bar plots (symbol: mean value; whiskers: minimum and maximum value) of centering ratios for the different instrument systems used (calculations aggregated for all canal configurations). Middle: Error bar plots of centering ratios (calculations aggregated for all preparation systems). Right: Error bar plots of preparation times for the different preparation instruments. **B** Number of ledges created per canal configuration by the different preparation instruments.

F6 SkyTaper #25 had the best overall centering ratio (mCR 0.60 (SD 0.067)) of all preparation systems calculated as mean centering ratio from all different canal configurations. This was significantly (p<0.001) better compared to the other systems apart from OneShape (mCR 0.58 (SD 0.086)) and F360 #25 (mCR 0.57 (SD 0.110)) (Fig 3A).

**Fig 4:**
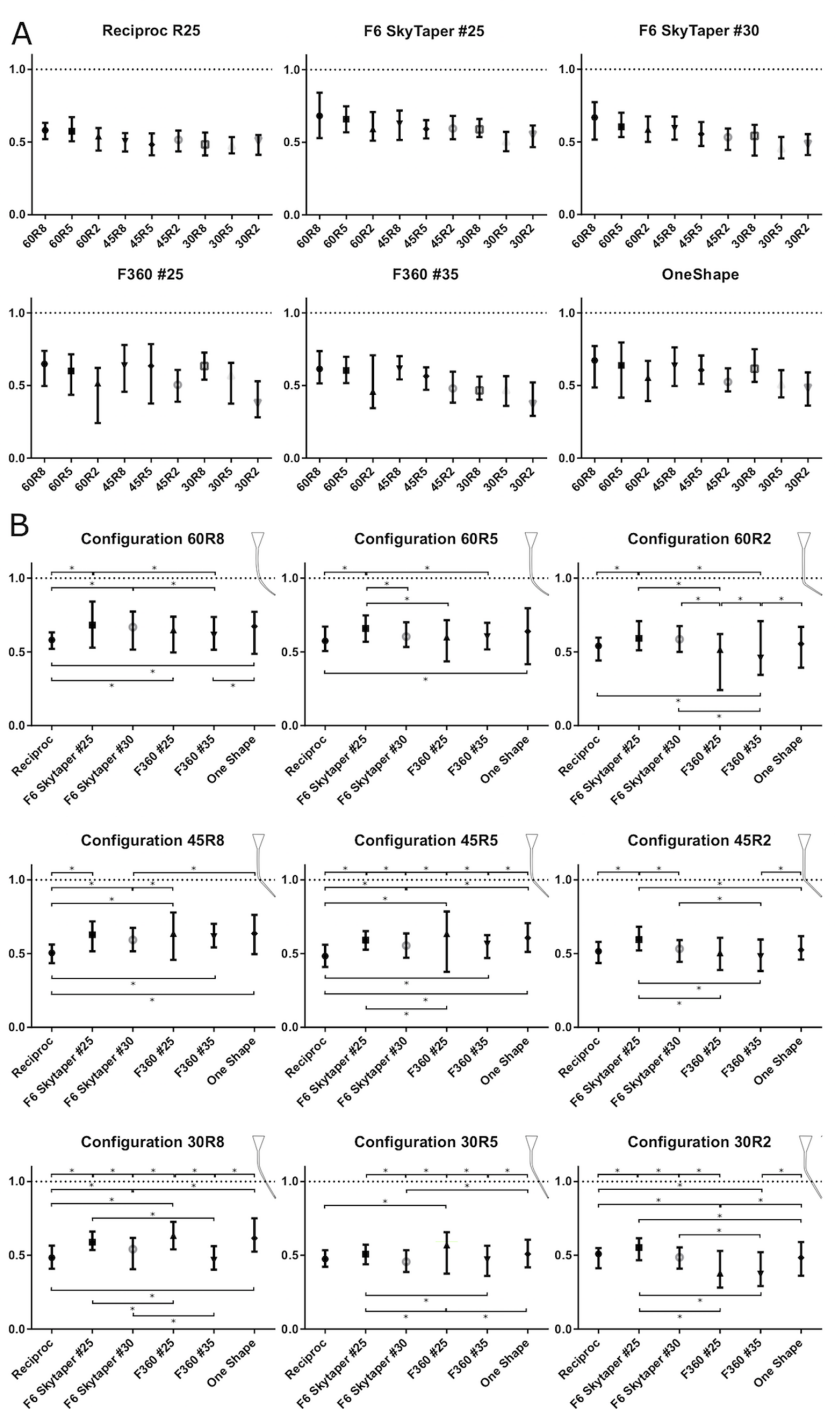
**A** Error bar plots (symbol: mean value; whiskers: minimum and maximum value) of mean centering ratios for the different instrument systems used for the different root canal configurations. **B** Error bar plots of mean centering ratios for the different canal configurations with the different instrument systems. (Asterisks indicate significant differences (p<0.05).

The Reciproc R25 file stayed best centered in the canal configuration with a curvature angle of 60° and a curvature radius of 8 mm (60R8) (mCR 0.58 (SD 0.031)) and was least centered in the configuration 30R5 (mCR 0.48 (SD 0.041)). The difference between best and least centered configuration was significant (p<0.0001) (Fig 4A).

The F6 SkyTaper #25 file stayed significantly (p<0.001) best centered in the 60R8 configuration compared to the other configurations except from the 60R5 which was the best mCR (0.68 (SD 0.076)) of all systems used in this study. The file was significantly (p<0.01) less centered in 30R5 compared to the other canal configurations (Fig 4A).

The F6 SkyTaper #30 file had a similar preparation profile to the F6 #25. The file was significantly (p<0.0001) best centered in 60R8 (mCR 0.67 (SD 0.061)) and significantly least centered in 30R5 (mCR 0.46 (SD 0.037)) (Fig 4A).

The F360 #25 file was best centered in all configurations with a radius of 8mm without statistical significant differences (p>0.99). The file was significantly (p<0.0001) least centered in the 30R2 configuration (mCR 0.38 (SD 0.068)) compared to all other canal configurations (Fig 4A).

The F360 #35 file was best centered in the 45R8 configuration (mCR 0.61 (SD 0.043)). In the 30R2 configuration it was significantly (p<0.001) least centered (mCR 0.37 (SD 0.051)) compared to the other canal configurations (Fig 4A).

OneShape had the best mean centering ratio in the 60R8 (mCR 0.67 (SD 0.063)) configuration and was least centered in the 30R2 configuration (mCR 0.48 (SD 0.059)) (Fig 4A).

### Evaluation of the different canal configurations

Generally, the overall mean centering ratio calculated combined for all preparation systems was significantly (p<0.001) better in the root canal configuration with a curvature angle of 60° and a curvature radius of 8mm (60R8) compared to all other configurations (Fig 4B). The significantly (p<0.001) least centered overall preparations were found in the 30R2 canal configuration. Generally, the preparations were better centered in canal configurations with higher curvature angles and higher radii.

**Fig 5:**
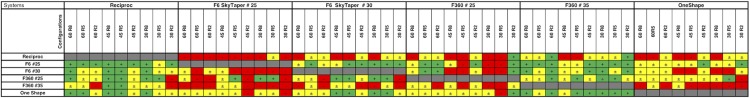
Overview of the significant differences between the systems in the different canal configurations. Green fields indicate a significant (p<0.05) better centering ratio of the file system in lines compared to the file in the specified canal configuration in the corresponding column. Red fields indicate significant (p<0.05) lower centering ratios and yellow fields indicate no significant difference.

While F6 SkyTaper #25 had the best mean centering ratio in four out of 9 canal configurations, Reciproc had the worst mean centering ratio in four configurations. Reciproc was not centered best in any configuration compared to the other systems. Fig 5 provides a quick comparison between the different file systems in the various canal configurations.

### Preparation time

Reciproc had a mean preparation time of 103.0 s (SD 20.67 s) and was significantly slower than all other systems (p<0.0001). OneShape had a mean preparation time of 86.7 s (SD 13.53 s) and was significantly (p<0.0001) faster than all other systems. F6 SkyTaper (92.1 s (SD 6.64 s)) and F360 (92.2 s (SD 12.93 s)) differed significantly from the other systems (p<0.0001) but not from each other (p>0.9999) (Fig 3A).

### Preparation faults and instrument fractures

During the preparation of the root canals 15 ledges occurred. 12 of them happened in the 60R2 and three in the 45R2 configuration. During root canal preparation one OneShape instrument fractured (Fig 3B).

## Discussion

In this study, it was possible to compare the shaping abilities of three rotary and one reciprocating root canal preparation system in a variety of different root canal configurations. The 3D-printing production method allowed creating different, systematically designed root canal configurations with increasing demands on the root canal preparation instruments. Even root canal configurations with challenging anatomies could be tested. We observed a tendency that anatomies with small curvature radii and small curvature angles led to lower centering ratios. The results for the overall centering ratios of the different canal configurations show a greater influence of the curvature radius than the curvature angle. The canal configurations that were tested in our study showed a better centering ratio in cases with greater curvature angles. We explain the lower centering ratios in root canal anatomies with smaller curvature angles of 30° with the fact that the starting-point of the curvature was more coronally in these cases than in cases with greater curvature angles. Because root canal instruments are tapered and therefore stiffer in direction of the shaft, more transportation will result in cases with more coronally located curvatures. In the literature, many studies can be found in which root canal instruments were tested in simulated root canals [1, 7–9, 11, 14–16, 18]. Clear resin blocks are therefore a widely used method that is thought to be highly reproducible. While the method is widespread, there is no detailed information on the canal configurations that were used. In most studies, only the shape of the canal configuration (J- or S-shaped) and the manufacturer [9, 18, 30] and sometimes also the curvature angle is mentioned [14]. To the knowledge of the authors, only one study states curvature angle and curvature radius [31]. One problem is that different authors suggested different methods to measure these values [28, 32, 33]. The other problem is that measuring these values is unreliable even when performed by experienced endodontists and should be better done with the help of software tools [34]. It is also difficult to state these values because the production related differences between resin blocks [22] would make it necessary to measure each block individually, contradicting the idea of a high reproducibility of this experimental design. Already in former unpublished studies we experienced that there are indeed differences between resin training blocks even of the same batch making it impossible to work with measuring templates, although considerable efforts have already been made to make measurements more reproducible and less prone to subjectivity of an investigator through the use of digital technologies [19–21]. To avoid the use of templates we originally developed the described software measuring tool to minimize measuring faults related to manual measurement methods with templates. Moreover, the acrylic resin blocks used in this study were products of stereolithographic 3D-printing. Therefore, it was possible to design root canal configurations as demanded.

To the knowledge of the authors there is only one study that investigated the effect of different root canal anatomies on the outcome of root canal preparation with different instruments [35]. The study was conducted with roots of extracted teeth that were classified as straight, J- and C-shaped canals. In this study the type of canal configuration had a considerable impact on the asymmetry of root canal preparation. Curved canals showed significantly more asymmetrical preparations than straight canals. The instrument type also had an influence on the symmetry of the preparations [35]. The instruments used were either stainless steel hand instruments or engine driven stainless steel instruments, which is a considerable difference to our study. We also found an impact of the canal configuration on the centering ratio. Generally, canal configurations with a big curvature angle and radius showed the best centering ratio, while configurations with a small curvature angle and radius showed the least centered preparations.

Regarding the centering ability, no single system was generally superior to the others in all canal anatomies. Best centered preparations were achieved with F6 SkyTaper #25 instruments in 60R8 anatomies, while the least centered preparations were achieved with F360 #35 in 30R2 anatomies. On the one hand, this can be explained by the fact that the F360 #35 had the biggest ISO size of all instruments and was therefore the stiffest file tested in our study at least at the instrument tip. On the other hand, by the fact that anatomies with greater curvature angles and radii exhibited better centered preparations than anatomies with small curvature angles and radii. It is remarkable that the F360 and OneShape instruments were the least centered instruments in all canal configurations with a radius of 2 mm, while F6 SkyTaper and Reciproc instruments showed more homogenous centering ratios in all configurations that were tested. This finding can only be partially explained. It may not be very surprising that Reciproc instruments generally have a tendency towards less centered preparations because these instruments had the greatest taper of all instruments in this study. The resulting greater stiffness of the instrument will therefore lead to more transportation. But this does not fully explain why the F360 and OneShape instruments with smaller tapers had no better centering ratios than Reciproc in cases with a curvature radius of 2 mm. However, F360 and OneShape instruments seem to be less suitable for root canals with abrupt curvatures than the other instruments.

Other studies certified that all systems of our study maintain the original canal curvature well without significant differences [25, 27, 36]. These studies were done on extracted teeth with curvature angles of about 29–33° and radii of about 6.5–7.5mm. This would be roughly equivalent to our configurations of 30R8 and 30R5 in which we found significant differences between the preparation systems. Perhaps this can be explained by the fact that acrylic resin has a lower hardness than dentine [37] and therefore shows more pronounced differences between the instrument systems.

Another selection criterion for root canal instruments is the incidence of preparation faults. In our study, most ledges were created in the 60R2 canal configuration. That greater curvature angles lead to a higher frequency of ledges is a common finding in different publications [38–40]. In the present study we found that it was not the curvature angle alone that led to a higher incidence of ledges but the combination with a small curvature radius. Because the 60R2 configuration has the most abrupt curvature, this anatomy seems to be a considerable challenge for most of the root canal instruments tested in our study. Therefore, root canal anatomies with abrupt curvatures seem to have a greater influence on the incidence of ledges in our study than the instrument type. Regarding the instrument type we found that only with F6 SkyTaper #25 and F360 #25 instruments no ledges occurred. Perhaps this can be explained because F360 #25 instruments had the smallest taper of only 0.04 and were the most flexible instruments in this study with a lower risk of producing ledges. Interestingly, with increased ISO size, F6 SkyTaper #30 and F360 #35 also produced ledges. Therefore, it may be sensible to dispense with larger preparation diameters to avoid ledge creation. Most ledges occurred with OneShape instruments (Fig 3B). Perhaps this can be explained because the triangular cross-shape geometry at the instrument tip lowers the flexibility of the OneShape instrument compared to the other instruments with a S-shaped geometry.

In this study OneShape had the fastest mean preparation times of about 86 s. The slowest mean preparation times were achieved with Reciproc (103 s). This is in contrast to other in-vitro studies that reported preparation times of 73–75 s for Reciproc and of 80–114 s for OneShape [25, 27, 36]. The reason for this study outcome is difficult to explain. We can only speculate that perhaps the acrylic resin material has an impact on the cutting efficiency of reciprocating instruments which is in contrast to cutting natural dentine that has been used as experimental model in the abovementioned studies.

Moreover there could be an unknown impact of ethanol as irrigation solution, maybe altering the properties of the resin material of the root canal models and also the behavior of the different root canal instruments. But during our pre-studies we made the practice based experience, that in our acrylic resin blocks EDTA and NaOCl did not work in the same way as lubricant as it normally works in dentine. Therefore we tried other solutions for irrigation and found that ethanol was more suitable. Moreover, in a lot of the studies involving resin blocks, either water or different types of alcohols are used as irrigation solutions [14, 15, 18–21, 31]. To the knowledge of the authors there are no publications describing the exact reason for the use of different types of irrigations. Therefore the impact of the irrigant on the performance of the instruments cannot be estimated.

## Conclusion

Stereolithographic 3D-printed root canal models open the opportunity to produce challenging canal configurations to systematically investigate the capabilities and the limitations of root canal instruments. Root canal configurations with small curvature radii led to low centering ratios and increased number of ledges. All NiTi files caused canal transportations. No single system was totally superior to the others. Best centered preparations were achieved with F6 SkyTaper #25 instruments. Reciproc was significantly slower than all other systems and OneShape fastest. One OneShape instrument fractured and these instruments produced most ledges.

## Supporting information

S1 File Source code. Source code as cpp File.
(CPP)

S2 File Source code. Source code as txt File.
(TXT)

S3 File Compressed Executable + Source Code Executable + Source Code. (ZIP)
